# Effects of predictive nursing intervention on cognitive impairment and neurological function in ischemic stroke patients

**DOI:** 10.1002/brb3.2890

**Published:** 2023-02-03

**Authors:** Lianyu Xue, Jiangshan Deng, Lingyan Zhu, Feifei Shen, Jiewei Wei, Lihui Wang, Qinqin Chen, Lan Wang

**Affiliations:** ^1^ Department of Nursing Shanghai Sixth People's Hospital Affiliated to Shanghai Jiao Tong University School of Medicine Xuhui District, Shanghai China; ^2^ Department of Neurology Shanghai Sixth People's Hospital Affiliated to Shanghai Jiao Tong University School of Medicine Xuhui District, Shanghai China; ^3^ Department of Gastroenterology Shanghai Sixth People's Hospital Affiliated to Shanghai Jiao Tong University School of Medicine Xuhui District, Shanghai China

**Keywords:** cognitive impairment, complications, ischemic stroke, neurological function, predictive nursing intervention, thrombolytic therapy

## Abstract

**Background:**

Ischemic stroke is a clinical emergency caused by insufficient intracranial blood supply, which eventually leads to brain tissue necrosis and neurological impairment. Predictive nursing intervention has achieved impressive success in the nursing of multiple surgeries. However, the role of predictive nursing intervention in the care of patients with ischemic stroke remains unclear.

**Methods:**

This study was a randomized controlled trial. Based on the inclusion and exclusion criteria, 126 patients were randomly assigned into two groups, namely the control group and the predictive nursing intervention group. Both groups were treated with thrombolytic therapy with alteplase. The patients in the control group were given routine nursing intervention and the predictive nursing intervention group received additional predictive care. Neurologic functions and cognitive impairment were evaluated by National Institutes of Health Stroke Scale (NIHSS), Fugl‐Meyer assessment (FMA), Montreal cognitive assessment (MoCA), and mini‐mental state examination (MMSE) scales, respectively. Door‐to‐Needle Times, venous thromboembolism (VTE)‐related parameters, and complications were recorded.

**Results:**

Predictive nursing intervention significantly shortened the Door‐to‐Needle Times and enhanced the peak/average femoral venous blood flow and femoral venous diameter. In addition, predictive nursing intervention improved the NIHSS, FMA, MMSE, and MoCA scores and remarkably reduced the recurrence of ischemic stroke, deep vein thrombosis and gingival bleeding.

**Conclusion:**

Predictive nursing intervention is beneficial to improve the effects of thrombolytic therapy in patients with ischemic stroke, which improves the neurological, cognitive and motor functions of patients, and reduces the occurrence of complications, suggesting an important clinical application value.

## Graphical abstract

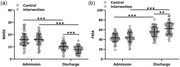

## 1 INTRODUCTION

Ischemic stroke (cerebral infarction) is mostly due to thrombosis, lack of anticoagulant, surgery and other reasons that lead to obstruction of cerebral blood flow, resulting in symptoms such as local hypoxic‐ischemic lesions and neurological deficits (Clark et al., 2014; Rabinstein, 2020). Acute cerebral infarction has a high morbidity rate, high disability rate and high fatality rate, which seriously endangers the physical and mental health of patients and their families (Herpich & Rincon, 2020; Hinkle & Guanci, 2007).

Common clinical treatments for ischemic stroke include thrombolytic therapy, antiplatelet therapy, interventional therapy, and surgery (Mendelson & Prabhakaran, 2021). Through clinical practice research, it is found that intravenous thrombolysis within the time window is one of the effective ways to treat acute cerebral infarction (Silva & Nogueira, 2020). The thrombolytic time window refers to the thrombolytic therapy by administering recombinant tissue plasminogen activator (r‐tPA, alteplase) within 4.5 h of the onset of the patient (Jolugbo & Ariens, 2021). The therapeutic effect depends on the time of thrombolysis (Ho, 2021). The earlier the treatment is performed, the better the effect may be, which could effectively reduce the mortality rate of patients (Grotta, 2021). During the course of thrombolytic therapy, patients often suffer from a series of symptoms such as lower extremity edema and pain due to prolonged bed rest and limited mobility of the lower limbs, which seriously affects the prognosis of the patients (Qiu et al., 2021). On the other hand, when alteplase is used to treat acute stroke patients, complications such as hemorrhage, allergic reaction, and epilepsy may occur, which will affect the recovery effect of patients (Boling & Groves, 2019; Ospel et al., 2020). Therefore, it is necessary to strengthen nursing intervention.

Predictive nursing intervention means that before the formal implementation of nursing operations (Cook et al., 2020), nurses conduct comprehensive analysis of possible risks in the process of treatment and predictive analysis of problems that may occur in the nursing process, and formulate corresponding nursing procedures to avoid and reduce the incidence of risk (Barch et al., 1997; Dias & Figueiredo, 2015). Studies have shown that effective nursing intervention can improve the therapeutic effect of intravenous thrombolysis in acute cerebral infarction and reduce the occurrence of complications (Butt et al., 2021; Qin et al., 2008). Predictive nursing intervention could comprehensively evaluate and analyze the patient's condition and make advance judgment on risk factors (Brown et al., 2016). Therefore, this study aimed to investigate the effect of predictive nursing intervention on neurological function and complications in ischemic stroke patients received thrombolytic therapy.

## 2 METHODS

### 2.1 Patients

A total of 126 patients diagnosed with acute ischemic stroke and met the inclusion and exclusion criteria in our hospital were selected as the test subjects. The patients were randomly divided into the control group and the predictive nursing intervention group by the random number table method, with 63 cases in each group. Briefly we used a special shaker to automatically roll out a number one by one to make a table for future reference. The chance of any number in this list is equal. All participants signed written informed consent. The study was approved by the ethics committee of Shanghai Sixth People's Hospital Affiliated to Shanghai Jiao Tong University School of Medicine.

Inclusion criteria: (1) those who met the diagnostic criteria in “China Guidelines for Diagnosis and Treatment of Acute Ischemic Stroke 2018”; (2) CT and MRI examinations of the brain meet the diagnostic criteria for acute ischemic stroke; (3) first onset, acute onset, onset time ≤ 4.5 h; (4) those who met the indications for thrombolytic therapy; (5) expected survival time ≥ 3 months; (6) no coagulation dysfunction; no history of drug allergy; no severe liver and kidney damage; no aphasia, hearing impairment, etc.; (7) who were able to read and understand the informed consent form.

Exclusion criteria: (1) those who have diseases such as abnormal immune system and coagulation dysfunction; (2) those who have traumatic brain injury, hemorrhagic stroke, cerebral vascular malformation, epilepsy; (3) those who have history of intracranial hemorrhage; (4) those who have experienced gastrointestinal and urinary system bleeding in the past 3 weeks; (5) those who have undergone arterial puncture in the past 7 days; (6) those who have received anticoagulant therapy; (7) those who have previous cognitive, mental, and communication impairments and cannot cooperate with this researcher.

Patients in both control group and intervention group received thrombolytic therapy with alteplase at a dose of 0.9 mg/kg (maximum dose 90 mg). Ten percent of the total dose of the drug was injected intravenously within 1 min, and the remaining 90% was intravenously pumped at a uniform rate within 1 h. The patient's vital signs need to be closely monitored and the patient's blood pressure should be monitored every 15 min at 2 h after thrombolytic therapy, t. Head CT was performed to rule out intracranial hemorrhage, and aspirin 200 mg/day was administered orally at 24 h after thrombolysis. If the patient has intracranial hemorrhage, various conventional treatments such as anti‐arteriosclerosis should be carried out in time and attention should be paid to improving cerebral circulation.

### 2.2 Grouping

The patient allocation sequence was generated using a complete randomization procedure. The random number and the associated allocated treatment were kept in sequentially numbered sealed opaque envelopes.

Patients in the control group received traditional usual care in accordance with our hospital's usual care process:

Sign monitoring: After the patient is admitted to the hospital, the patient's physiological signs, such as blood pressure, heart rate, respiratory rate and other indicators should be regularly monitored every day, and the patient's specific condition should be evaluated. If the patient has symptoms such as increased blood pressure, dyspnea, infection, thrombosis, etc., the attending doctor should be notified in time and corresponding treatment measures should be taken.

Routine nursing: During the patient's bedridden period, the patient's body position should be changed regularly, the respiratory secretions should be cleaned up in time, and the breathing should be kept smooth. The patients should be told to drink more water and eat more food rich in protein and vitamins. If the patient has difficulty urinating, a catheter can be placed to assist in urination.

Health education: Patients should be taught disease‐related knowledge, such as pathogenesis, treatment methods, treatment precautions, and common adverse drug reactions. The patient's psychological mood should be paid attention to, and patients with psychological abnormality should be counseled in time. Nursing should be closely communicated with family members to encourage family members to support and encourage and to reduce the psychological burden of patients.

Rehabilitation training: Before being discharged from the hospital, the patients were assisted to complete the rehabilitation training, and were given discharge guidance and rehabilitation guidance. The patient could be discharged from the hospital after he/she could complete the training independently.

Follow‐up: The patients were followed up by telephone every month after discharge to investigate the recovery of the patients.

The predictive nursing intervention group implements predictive care on basis of usual care in the control group, including the following:

Predictive nursing for allergic reactions: The dose of alteplase was precisely configured before thrombolysis, and the infusion rate was adjusted as required by the doctor. Symptoms and vital signs during thrombolysis should be closely observed. If the patient has an allergic reaction, the infusion of alteplase should be stopped, the doctor should be informed in time, and professional treatment should be carried out with antihistamines, glucocorticoids, etc. as prescribed by the doctor.

Predictive nursing of bleeding: The bleeding risk of patients should be assessed before thrombolysis to understand the degree of risk. For patients with higher risk, the thrombolysis time should be shortened as appropriate. During thrombolysis, the patient's blood pressure should be monitored dynamically. If systolic blood pressure > 185 mmHg, diastolic blood pressure > 110 mmHg, the thrombolysis should be suspended. Two hours after thrombolysis, the patient should be instructed to stay in bed absolutely, pay attention to the gentleness and flexibility of the movements when changing the position, avoid sharp turning of the head, and strengthen respiratory care to ensure that it is in a smooth state.

Predictive nursing for reperfusion brain injury: Within 24 h after thrombolysis, the patient's consciousness, blood pressure, breathing and other indicators should be closely monitored. On the basis of objectively assessing the remission of the patient's original condition, the 24‐h intake and output were accurately recorded, and diet and drinking instructions were given to maintain a balance of the 24‐h intake and output. In addition, if the patient has fever symptoms, it is necessary to inform the doctor in time, and cooperate with the doctor to cool down through physical methods.

Predictive nursing for vascular reocclusion: During the thrombolysis process, the patient's neurological deficit dynamic score was given, a complete set of coagulation and blood routine review was performed every 6 h, and platelets and clotting time were regularly monitored to determine whether the patient has vascular reocclusion risk, and aspirin intervention should be prescribed.

Psychological predictive nursing: After admission, patients and their families are prone to anxiety, tension, and fear. Therefore, nursing staff should actively introduce the hospital environment, attending doctors, and bed nurses to patients and their families, and help patients to do inspections and assessments. Nursing staff should fully understand the patient's family background, cultural background, economic status, main manifestations before and after the illness, and reevaluate according to their psychological state. Nursing staff should explain the main methods, importance and significance of thrombolytic therapy to patients, relieve patients’ anxiety and tension after admission, and at the same time enhance patients’ confidence in thrombolytic therapy, so as to relieve patients’ high blood pressure caused by emotional changes and prevent the occurrence of brain hemorrhage.

Predictive nursing for deep vein thrombosis of lower extremities: due to factors such as bed rest and slow blood flow after surgery, venous thrombosis of lower extremities may be caused. Therefore, after thrombolytic therapy, it is necessary to observe whether the dorsal foot artery of the patient is weakened or disappeared, whether the skin color and skin temperature of the lower extremity are normal, and ask whether the patient has pain and sensory disturbance in the lower extremity. At the same time, nursing staff should pay attention to the fact that the puncture site should not be bandaged too tightly to prevent the arterial blood supply from being affected. Once the limbs are pale, the calf is numb, pain, the skin color turns black, and the dorsal artery pulse disappears, it indicates that the patient may have lower extremity arterial thrombosis. Nursing staff should immediately contact the bed doctor, do blood vessel color Doppler ultrasound, and carry out follow‐up treatment according to the diagnosis results.

Predictive nursing of infection: For patients who need to indwelling catheters, nursing staff should choose appropriate catheters, strictly perform aseptic operations during indwelling, replace urine collection bags in time, and disinfect catheter openings every day to prevent urinary tract infections happened. Nursing staff should strengthen the patient's oral care, instruct the patient to rinse the mouth after eating, and do a good job of oral hygiene every day. If there is a swallowing disorder, the nursing staff should instruct the patient to sit as much as possible when eating, take a liquid diet, and eat small and frequent meals to prevent the occurrence of aspiration.

### 2.3 Neurologic function assessment

Neurologic function was evaluated by National Institutes of Health Stroke Scale (NIHSS) and Fugl‐Meyer assessment (FMA).

NIHSS includes consciousness, language, motor function, sensory deficits, visual field deficits, eye movements, coordinated movements, neglect, and articulation, etc. The total NIHSS scores were 42. Scores of 0−1 for normal status, 2−4 for mild neurologic impairment, 5−15 for moderate neurologic impairment, 16−20 for severe neurologic impairment, and 21−42 for extremely severe neurologic impairment.

FMA is an ordinal scale with 3 scores for each item. A score of 0 was given if the subject was unable to complete a task, 1 was given for partial completion, and 2 was given for complete completion. However, there were only two scores for reflex activity, 2 and 0 for the presence and absence of reflex activity, respectively. The five areas of FMA assessment are motor function (up to 66 for upper body and 34 for lower body); sensory functions (up to 24); balance ability (up to 14); joint range of motion (up to 44); joint pain (up to 44). A maximum of 226 points could be obtained for the FMA assessment; it is common practice to assess all areas separately, and the test lasts approximately 40 min. For the test, the inspector needs a tennis ball, a small ball container and a knee hammer.

### 2.4 Cognitive impairment assessment

Cognitive impairment was evaluated by Montreal cognitive assessment (MoCA) and mini‐mental state examination (MMSE) scales. MoCA includes cognitive domains such as attention and concentration, memory, executive function, visual structural skills, language, abstract thinking, calculation, and orientation. The total score is 30 points, and 26–30 points are normal. MMSE includes immediate memory, time‐oriented force, location‐oriented force, delayed memory, language, visual space, attention and calculation, etc. The total score is 30 points, 27–30 is normal, 21–26 is mild mental retardation, 10–20 points is moderate intellectual disability, 0–9 points is severe intellectual disability. The intragroup correlation coefficient of the MMSE joint examination was .99, and the MoCA scale Cronbach's alpha was .83.

### 2.5 Statistical analysis

SPSS19.0 software was used for statistical analysis and data was expressed as mean ± SD. Values were expressed as *n* (percentage, %) or mean ± SD. For comparing two groups, Mann–Whitney test was used. For multiple comparisons tests, two‐way ANOVA followed by Tukey's was used. Chi‐square test or Fisher's exact test was used for assessing distribution of observations or phenomena between different groups.

## 3 RESULTS

### 3.1 Study design

**FIGURE 1:**
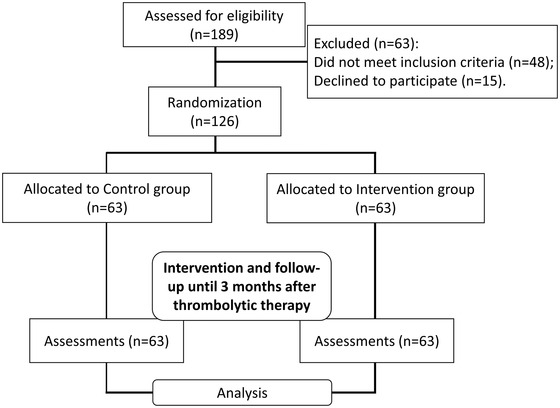
Research framework of this study.

The flowchart of the whole experiment was shown in Figure 1. After assessed for eligibility, 189 ischemic stroke patients were initially enrolled in the study. Next, 63 patients were excluded from the study, including 48 patients who did not meet the inclusion criteria and 15 patients declined to participate. Therefore, a total of 126 patients were randomly assigned into two groups, namely the control group (*n* = 63) and the predictive nursing intervention group (*n* = 63). Both groups were treated with thrombolytic therapy with alteplase. The patients in the control group were given routine nursing intervention. The predictive nursing intervention group implemented predictive care on the basis of routine nursing intervention. Follow‐up was performed until the end of the trial three months after thrombolysis, and then data analysis was performed.

### 3.2 Demographic and clinical characteristics of the study participants

The baseline data of the two groups were shown in Table 1. No significant difference was observed in age, gender, BMI, time from onset to admission, hemoglobin, blood platelet count, blood glucose, blood pressure, stroke classification, smoking history, diabetes history, hypertension history, and coronary heart disease between two groups (all *p* > .05).

**TABLE 1:** Demographic and clinical characteristics of the ischemic stroke patients received thrombolytic therapy

	Study group		
Characteristics	Control (*n* = 63)	Intervention (*n* = 63)	*p* Value
Age (years)	63.7 ± 8.2	64.4 ± 9.4	.263
Gender			
Male	36 (57.1%)	41 (65.1%)	.471
Female	27 (42.9%)	22 (34.9%)	
BMI (kg/m^2^)	24.7 ± 4.2	24.9 ± 5.1	.496
Time from onset to admission (h)	2.75 ± 0.79	3.03 ± 0.87	.223
Hemoglobin (g/L)	138.42 ± 21.9	146.17 ± 26.4	.174
Blood platelet count (10^9^/L)	173.86 ± 38.5	165.92 ± 42.77	.206
Blood glucose (mmol/L)	7.05 ± 2.48	6.69 ± 2.68	.149
Systolic blood pressure (mmHg)	148.52 ± 20.83	153.16 ± 18.47	.277
Diastolic blood pressure (mmHg)	89.45 ± 14.74	87.83 ± 16.18	.304
Stroke classification			
Cerebral thrombosis	37 (58.7%)	33 (52.4%)	.552
Cerebral embolism	15 (23.8%)	14 (22.2%)	
Lacunar cerebral infarction	11 (17.5%)	16 (25.4%)	
Smoking history			
Yes	35 (55.6%)	30 (47.6%)	.476
No	28 (44.4%)	33 (52.4%)	
Diabetes history			
Yes	14 (22.2%)	16 (25.4%)	.835
No	49 (77.8%)	47 (74.6%)	
Hypertension history			
Yes	24 (38.1%)	29 (46%)	.471
No	39 (61.9%)	34 (54%)	
Coronary heart disease			
Yes	15 (23.8%)	19 (30.2%)	.548
No	48 (76.2%)	44 (69.8%)	

*Note*: Values were expressed as *n* (percentage, %) or mean ± SD. *p* Values for each group were derived from Mann–Whitney test. Chi‐square test or Fisher's exact test was used for assessing distribution of observations or phenomena between different groups.

BMI: body mass index.

### 3.3 Effects of predictive nursing intervention on Door‐to‐Needle Times and venous thromboembolism (VTE)‐related parameters

**FIGURE 2:**
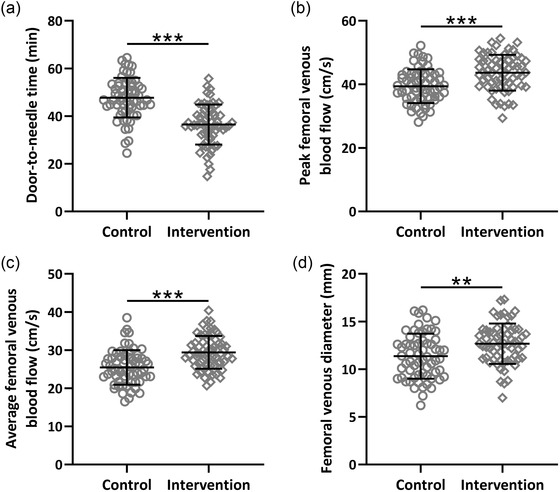
Effects of the predictive nursing intervention on Door‐to‐Needle Times (a), peak femoral venous blood flow (b), average femoral venous blood flow (c), and femoral venous diameter (d) in ischemic stroke patients received thrombolytic therapy. Mean ± SD showing all the data. **p < .01, ***p < .001. Mann–Whitney test.

Door‐to‐Needle Times and VTE‐related parameters in the two groups of patients were analyzed. The results showed that predictive nursing intervention significantly shortened the Door‐to‐Needle Times (Figure 2a, *p* < .001) and enhanced the peak femoral venous blood flow (Figure 2b, *p* < .001), average femoral venous blood flow (Figure 2c, *p* < .001) and femoral venous diameter (Figure 2d, *p* < .01) in ischemic stroke patients received thrombolytic therapy.

### 3.4 Effects of the predictive nursing intervention on neurological function

**FIGURE 3:**
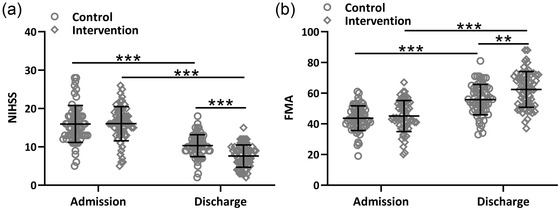
Effects of the predictive nursing intervention on neurological function in ischemic stroke patients received thrombolytic therapy. Comparisons of NIHSS (a) and FMA score (b) between the two groups at admission and discharge. Mean ± SD showing all the data. **p < .01, ***p < .001. Two‐way ANOVA followed by Tukey's multiple comparisons tests.

The NIHSS and FMA were used to analyze the neurological function scores of the two groups at admission and discharge. We found that there was no significant difference in the NIHSS and FMA scores between the two groups of patients at admission. At discharge, the NIHSS and FMA scores improved in both groups, whereas the predictive nursing intervention group improved more significantly (Figure 3a and b).

### 3.5 Effects of the predictive nursing intervention on cognitive impairment

**FIGURE 4:**
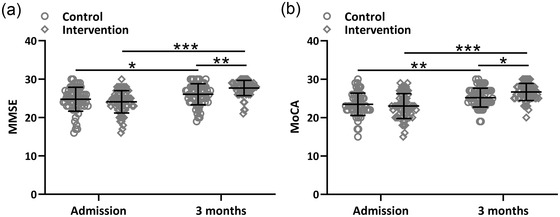
Effects of the predictive nursing intervention on cognitive impairment in ischemic stroke patients received thrombolytic therapy. Comparisons of MMSE (a) and MoCA (b) between the two groups at admission and 3 months after thrombolytic therapy. Mean ± SD showing all the data. *p < .05, **p < .01, ***p < .001. Two‐way ANOVA followed by Tukey's multiple comparisons tests.

The MMSE and MoCA were used to analyze the cognitive impairment of the two groups at admission and three months after thrombolytic therapy. We found that there was no significant difference in the MMSE and MoCA scores between the two groups of patients at admission. Three months after thrombolytic therapy, the MMSE and MoCA scores improved in both groups, whereas the predictive nursing intervention group improved more significantly (Figure 4a and b).

### 3.6 Effects of the predictive nursing intervention on complications during 3‐month follow‐up

After discharge, the patients were followed up by telephone every month to investigate the recovery of the patients, which lasted for three months. In Table 2, we compared the complications of the two groups of patients, and found that the predictive nursing intervention remarkably reduced the total occurrence of the complications (*p* = .007). Among the indicators, there were significant differences in recurrence of ischemic stroke (*p* = .029), deep vein thrombosis (*p* = .033) and gingival bleeding (*p* = .017).

**TABLE 2:** Comparisons of the complications between the two groups after thrombolytic therapy during 3‐month follow‐up

	Study group		
Characteristics	Control (*n* = 63)	Intervention (*n* = 63)	*p*
Recurrence	10 (15.9%)	2 (3.2%)	.029
Infection	9 (14.3%)	4 (6.3%)	.241
Deep venous thrombosis	8 (12.7%)	1 (1.6%)	.033
Pressure sores	7 (11.1%)	3 (4.8%)	.323
Intracranial hemorrhage	5 (7.9%)	0 (0%)	.058
Gastrointestinal hemorrhage	8 (12.7%)	2 (3.2%)	.095
Gingival bleeding	9 (14.3%)	1 (1.6%)	.017
Total occurrence	23 (36.5%)	9 (14.3%)	.007

*Note*: Values were expressed as n (percentage, %). *p* Value was derived from chi‐square test.

## 4 DISCUSSION

The condition of patients with ischemic stroke is changeable and complex, especially in elderly patients with degeneration of various organs, poor immunity, and poor tolerance to interventional surgery, which may lead to many postoperative complications and slow recovery of the body (Yi et al., 2021). In addition, patients often have anxiety and depression because of their own illness, body state and age, which is not conducive to recovery (Zubair & Sheth, 2021). Routine nursing focuses on complication care rather than prevention, lacks risk management and control, and does not pay enough attention to patient psychological intervention, making it difficult to meet the growing nursing needs (Nicolas‐Jilwan & Wintermark, 2021). Predictive nursing intervention is a nursing model that reduces the incidence of nursing adverse events by assessing risks and giving comprehensive preventive interventions based on influencing factors (Amatangelo & Thomas, 2020). At present, this nursing model has been applied in various departments in China, and it has been reported that it can prevent deep vein thrombosis of lower extremities, postpartum depression, and constipation after stroke after orthopedic surgery. In this study, predictive nursing was used in patients with ischemic stroke who underwent interventional surgery, and good outcomes were obtained, and the analysis was based on the results of the study.

Early intravenous thrombolysis with r‐tPA in patients with acute cerebral infarction has become the main method of drug treatment for acute cerebral infarction (Kurminas et al., 2020). Thrombolysis time window is the key for patients with ischemic stroke to receive ultraearly treatment and reduce the mortality and disability rate (Liberale et al., 2020). The main complication of thrombolytic therapy is intracranial hemorrhage. When the indications and contraindications of thrombolysis are strictly controlled, the early intravenous thrombolysis of r‐tPA has a significant effect, and at the same time can reduce the occurrence of complications (Green et al., 2021; Osaadon et al., 2022). Some studies have suggested that active nursing could effectively shorten the treatment time of patients, while predictive nursing is to evaluate the possible risks and complications of patients in advance on the basis of active nursing, and take effective nursing measures in time (Sutherly et al., 2021; Yaghi et al., 2017). The results of this paper showed that when compared with the conventional nursing mode, predictive nursing shortened the DNT time in patients with ischemic stroke, prevented and reduced the occurrence of complications, and is of great significance for improving the treatment effect of the disease.

The neurology department of our hospital integrates predictive nursing intervention into the whole process of thrombolytic therapy, and the effect is remarkable. Predictive nursing means that nurses cooperate with doctors to quickly screen patients before thrombolysis, collect data, lead patients to complete CT examination, complete the collection of various blood samples, establish two effective intravenous channels, and complete all necessary procedures before thrombolysis to minimize DNT time. Brain and heart health instructors cooperate with physicians to perform prethrombotic neurological and cognitive function scores and health education for patients and their families to eliminate the nervous state of patients, and to cooperate with doctors for diagnosis and treatment with the most trusting attitude. Our results showed that predictive nursing intervention significantly improved the NIHSS, FMA, MMSE, and MoCA scores, indicating that the self‐care ability and neurological recovery of the predictive nursing group were better than those of the control group, and the brain injury was relieved more obviously than the control group. The reason for consideration may be that predictive nursing realizes multidisciplinary nursing and individual nutritional support, which lays a foundation for the rehabilitation of the body, and early rehabilitation exercise can effectively stimulate the recovery of nerve function and improve the function of the body. Psychological intervention and health education in predictive nursing can further improve the patient's mentality, improve self‐care ability, reduce complications, and promote the improvement of body function.

The results of this study showed that the incidence of complications in the predictive nursing group was lower than that in the control group, indicating that the implementation of predictive nursing for stroke patients could effectively reduce the incidence of ischemic stroke recurrence, deep vein thrombosis and gingival bleeding, and improve satisfaction. A common complication of stroke is deep vein thrombosis of the lower extremity, and once deep vein thrombosis of the lower extremity occurs, it is very easy to cause pulmonary embolism, and even lead to death of the patient. In addition, deep vein thrombosis of the lower extremity can cause the loss of function of the lower extremity and have a serious impact on the patient's life. Routine nursing for patients, although scientific in method, lacks predictability, and does not prevent it based on the analysis of risk factors of lower extremity venous thrombosis. As a result, the complication rate is high, and patient recovery is slow and satisfaction is low. The results of this study showed that the peak femoral vein velocity, average blood flow velocity, venous diameter, and lower extremity venous patency score in the predictive nursing group were better than those in the control group. The results indicated that predictive nursing could effectively prevent venous thrombosis in patients with thrombolytic therapy, and could improve the coagulation function of patients and prevent bleeding. This may be due to health education and informing patients about the main causes of venous thrombosis, self‐care methods, etc. We believe that our predictive care has important clinical significance for the prevention and treatment of lower extremity deep vein thrombosis in stroke patients. On the one hand, it improves the patient's awareness of the disease and recovery, so that the patient trusts the nursing staff more and better cooperates with the nursing work; on the other hand, it allows the patient and their family members to take preventive measures, thereby further improving the effect of thrombolysis and nursing effect. Second, lower extremity exercises for patients are beneficial to venous blood return and prevent thrombosis. Nursing staff strengthen the dietary guidance for patients, instruct patients to avoid eating high‐sugar and high‐fat foods, and instruct patients to eat more fruits, vegetables and other foods containing vitamins and cellulose, which can reduce blood viscosity and prevent hyperlipidemia, thereby avoiding the occurrence of venous thrombosis. Finally, nursing staff focus on drug prevention for patients, monitor patients' coagulation function indicators, and use relevant anticoagulant drugs according to doctor's orders, which also plays a role in preventing lower extremity deep vein thrombosis and bleeding.

## 5 CONCLUSION

In conclusion, predictive nursing intervention is beneficial to promote the recovery of the patient's neurological function, improve the daily living ability of the patient, and reduce the occurrence of complications of the patient, which has certain clinical promotion value.

## CONFLICT OF INTEREST

All of the authors declare that they have no competing interests.

### PEER REVIEW

The peer review history for this article is available at https://publons.com/publon/10.1002/brb3.2890.

## Data Availability

Data could be achieved upon reasonable request to the corresponding author.
